# Optimizing the literature search: coverage of included references in systematic reviews in Medline and Embase

**DOI:** 10.5195/jmla.2023.1482

**Published:** 2023-04-21

**Authors:** Marita Heintz, Gyri Hval, Ragnhild Agathe Tornes, Nataliya Byelyey, Elisabet Hafstad, Gunn Eva Næss, Miriam Bakkeli

**Affiliations:** 1 marita.heintz@fhi.no, Head Librarian, Department of Library Services, Norwegian Institute of Public Health, Oslo, Norway.; 2 gyri.hval@fhi.no, Senior Adviser, Division for Health Services, Norwegian Institute of Public Health, Oslo, Norway.; 3 ragnhildagathe.tornes@fhi.no, Head Librarian, Department of Library Services, Norwegian Institute of Public Health, Oslo, Norway.; 4 nataliya.byelyey@fhi.no, Senior Librarian, Department of Library Services, Norwegian Institute of Public Health, Oslo, Norway.; 5 elisabetvivianne.hafstad@fhi.no, Senior Adviser, Division for Health Services, Norwegian Institute of Public Health, Oslo, Norway.; 6 gunneva.naess@fhi.no, Adviser, Division for Health Services, Norwegian Institute of Public Health, Oslo, Norway.; 7 miriam.bakkeli@fhi.no, Head Librarian, Department of Library Services, Norwegian Institute of Public Health, Oslo, Norway.

**Keywords:** Systematic reviews, literature search, databases, MEDLINE, Embase, cross-sectional study

## Abstract

**Objective:**

The aim of this study was to investigate if the included references in a set of completed systematic reviews are indexed in Ovid MEDLINE and Ovid Embase, and how many references would be missed if we were to constrict our literature searches to one of these sources, or the two databases in combination.

**Methods:**

We conducted a cross-sectional study where we searched for each included reference (n = 4,709) in 274 reviews produced by the Norwegian Institute of Public Health to find out if the references were indexed in the respective databases. The data was recorded in an Excel spreadsheet where we calculated the indexing rate. The reviews were sorted into eight categories to see if the indexing rate differs from subject to subject.

**Results:**

The indexing rate in MEDLINE (86.6%) was slightly lower than in Embase (88.2%). Without the MEDLINE records in Embase, the indexing rate in Embase was 71.8%. The highest indexing rate was achieved by combining both databases (90.2%). The indexing rate was highest in the category “Physical health - treatment” (97.4%). The category “Welfare” had the lowest indexing rate (58.9%).

**Conclusion:**

Our data reveals that 9.8% of the references are not indexed in either database. Furthermore, in 5% of the reviews, the indexing rate was 50% or lower.

## INTRODUCTION

There is a vast need for research in the health sector, and every day more is added to the evidence burden. Evidence-Based Practice (EBP) aims to provide the most effective care that is available by use of research evidence, along with clinical expertise and patient preferences. But for decision makers and health personnel, it can be challenging to identify the best available evidence. Evidence syntheses are aimed to aid this situation. By combining the results from multiple studies that have the same scope and object of study, the evidence synthesis offers a more complete picture of the research with a stronger conclusion than any single study can offer, due to the accumulated results. The former Norwegian Knowledge Centre for Health Services (NOKC), now a division in Norwegian Institute of Public Health (NIPH), delivers a considerable number of syntheses every year. Since the founding of the organization in 2004, they have produced hundreds of systematic reviews, health technology assessments (HTAs), overviews of reviews, and systematic mapping reviews on a broad spectrum of topics within medicine, health, and welfare services. High-quality reviews aim to identify as much evidence as possible that meets the inclusion criteria. Exhaustive literature searches in several sources are key to reaching this goal. The Cochrane Handbook emphasizes the need to conduct searches as extensively as possible [1] and, in the reviews produced by NOKC and NIPH, the usual practice is to search a wide range of databases [2].

Conducting comprehensive searches in multiple databases may be very time consuming and prolongs several steps in the review process. Information specialists performing the searches must familiarize themselves with the various search interfaces. The search strategies must be adapted to each database, with the correct use of search syntax, like truncations and proximity operators, before entering the search strategies into the different databases and running the searches. Search strategies from all databases must be documented and peer reviewed. Furthermore, performing searches in several databases leads to an increased number of references, all of which must be imported to a reference management program, deduplicated, and screened for inclusion. When aiming to identify as many eligible studies as possible, it is also common to complement the database searches with methods such as citation searching and reference checking. All of these are steps that add time to the review process. To make good decisions when it comes to use of resources we need more and updated studies that investigate where included studies are indexed, and the consequences of searching fewer databases than today's practice.

There are several issues that need to be explored before it's possible to determine the necessity of performing literature searches in line with the current recommendations, one of which is understanding the impact of searching a small number of databases. We can begin to investigate this by looking at finalized reviews where several databases were used in the literature searches. Checking how many of the included references are indexed in just one database or a select few databases tells us how many included studies we would miss out on, if we were to restrict our literature searches to these sources. Heath et al. [3] conducted a narrative review in which some of the included studies had the same aim as our study. Among these studies, we find Halladay et al. [4] and Frandsen et al. [5], which have gone through systematic reviews to see what proportion of included publications are indexed in MEDLINE. Halladay looked at 50 systematic reviews produced by Cochrane on therapeutic interventions, and Frandsen looked at all Cochrane reviews published between 2012 and 2016. Other noteworthy studies that have conducted similar work are Johansen et al. [6] and Mathisen [7]. Johansen looked at 104 systematic reviews conducted by EPOC (Cochrane Effective Practice and Organisation of Care) and Mathisen looked at 400 Cochrane reviews. The results from these studies ranged from a coverage of 70.9% [5] to a coverage of 93.64% [6], demonstrating inconsistent findings.

Similar to these studies, we initiated a cross-sectional study to investigate whether included references in a set of completed systematic reviews are indexed in MEDLINE and Embase, and how many references would be missed if we were to constrict our literature searches to one of these sources, or the two databases in combination. The systematic reviews, HTAs, overviews of reviews, and systematic mapping reviews (henceforth referred to as reviews) produced by NOKC and NIPH were chosen as the subject of investigation. All reviews produced since the organization's founding in 2004 to the current date were considered for inclusion.

MEDLINE and Embase were chosen because they are two leading databases in the field of medicine and health-related topics [8], which makes them natural choices for us if we were to search only one or two databases. Examining results from two databases gave us an opportunity to compare the results for the individual databases, which enabled us to make informed decisions on which database is preferable to choose if we were to search only one database. Measuring how Embase performs with and without MEDLINE records allowed us to see the potential impact of using Embase to search MEDLINE, rather than searching MEDLINE directly. Embase covers MEDLINE records by default, but it is possible to disregard these records by limiting the results to records copyrighted Elsevier B.V. only (limit x to conference abstracts or embase or “preprints (unpublished, non-peer reviewed)”). If choosing to search MEDLINE separately from Embase when conducting a literature search, applying this limitation makes sense. For this reason, we reported the results from Embase both with and without records from MEDLINE.

## METHODS

Two people from our team considered all publications published on the NIPH website [9] in the categories “Health technology assessment” and “Systematic review” published between 2004 and June 2020 for inclusion. To be included in our study, the review had to report: i) that MEDLINE and at least one additional database were searched, ii) a full search strategy for MEDLINE, iii) that two or more investigators independently screened the references found in the literature search, both by title/abstract, as well as the full text, and iv) the number of records identified, included and excluded in the review process in a flow diagram or in the running text. The included references from each review were copied into a Microsoft Excel spreadsheet by one team member and checked by another. We did not check if a reference was included in more than one review.

One team member attempted to retrieve each reference in MEDLINE and Embase using the following methods: i) title search, ii) title search with revised spelling or only a part of the title, iii) combining searches using journal name, year, volume, issue and/or pages, and iv) checking whether any articles from the journal in question are indexed in the database at all. Before concluding that a reference was not indexed in the databases, another team member made further retrieval attempts. The databases were searched using the Ovid interface. We used the most complete segment of Ovid MEDLINE, where unique PubMed records are retrievable (MEDLINE ALL). The results from Embase were recorded with and without the records from MEDLINE by checking the information in the copyright field in each record. To record whether a reference was indexed in the databases or not, we used four columns in the Excel spreadsheets: i) MEDLINE, ii) Embase (complete segment), iii) Embase (**©** Elsevier B.V. only), and iv) MEDLINE + Embase combined. When a reference was retrieved from a database, 1 was entered in the appropriate column. If a reference was not retrieved, 0 was entered.

We used embedded functions in Excel to sum the total number of retrieved references from MEDLINE alone, from Embase alone, with or without MEDLINE records, and the number of records retrieved in MEDLINE or Embase combined. We also calculated the percentage of coverage for each review, which we refer to as the indexing rate. The numbers for each review were copied into a separate spreadsheet where the reviews were sorted into eight main categories: i) Health systems and organization of care, ii) Health promotion and preventive medicine, iii) Physical health - treatment, iv) Physical health - diagnostics, v) Mental health - treatment, vi) Mental health - diagnostics, vii) Welfare services and, viii) Other. This was done in order to identify whether some areas had better coverage in the databases than others, as the reviews cover a wide variety of topics. The categories created for this paper reflect the organizational structure of the former Norwegian Knowledge Centre for Health Services (NOKC). A review could only be attributed to one category.

Additionally, we briefly checked the publication format and topic of the references that we could not retrieve from either database to identify reasons why these references were not indexed.

## RESULTS

In total, 274 reviews met our inclusion criteria, with a total of 4709 references to included studies. Table 1 shows included references in total and divided into each of the categories, in addition to the number of retrieved references in MEDLINE, Embase, and the databases combined. MEDLINE had a slightly lower average indexing rate (86.6%) than Embase (88.2%). When disregarding the MEDLINE records in Embase, the indexing rate dropped (71.8%). The highest indexing rate was achieved by combining both databases (90.2%). The results show that 2% of the included references would be lost by limiting the database choice to Embase. In the “Other” category, the results for MEDLINE alone are actually higher than Embase with MEDLINE records included.

**Table 1:** The indexing rate by category and database

Category (n of included systematic reviews)	No. of included references	MEDLINE	Embase (complete segment)	Embase (© Elsevier B.V. only)	MEDLINE + Embase combined
Health systems and organization of care (n=39)	604	565 (93.5%)	572 (94.7%)	482 (79.8%)	578 (95.7%)
Health promotion and preventive medicine (n = 47)	850	771 (90.7%)	772 (90.8%)	603 (70.9%)	783 (92.1%)
Physical health - treatment (n=84)	1366	1294 (94.7%)	1319 (96.6%)	1174 (85.9%)	1330 (97.4%)
Physical health - diagnostics (n=21)	216	195 (90.3%)	200 (92.6%)	149 (69.0%)	209 (96.8%)
Mental health - treatment (n=39)	535	457 (85.4%)	490 (91.6%)	402 (75.1%)	507 (94.8%)
Mental health - diagnostics (n=4)	108	91 (84.3%)	96 (88.9%)	72 (66.7%)	98 (90.7%)
Welfare (n=26)	457	252 (55.1%)	255 (55.8%)	191 (41.8%)	269 (58.9%)
Other (n=14)	573	452 (78.9%)	449 (78.4%)	309 (53.9%)	473 (82.5%)
Total (n=274)	4709	4077 (86.6%)	4153 (88.2%)	3382 (71.8%)	4247 (90.2%)

The number of references found varies between the different categories. The total indexing rate for the combination of MEDLINE and Embase was highest in the “Physical health - treatment” category (97.4%), while the “Welfare” category had the lowest indexing rate (58.9%).

**Figure 1:**
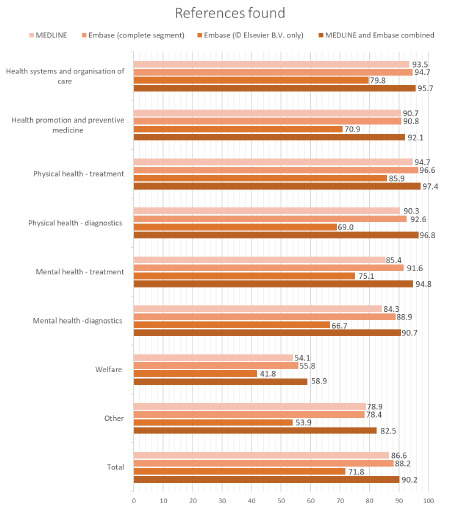
The indexing rate by category and database

In Figure 1, we can see the indexing rates across the different databases. The combination of MEDLINE and Embase has the highest percentage of references found in each of the categories, closely followed by Embase with the MEDLINE records included. In MEDLINE alone, we found slightly fewer of the references, and even fewer were found in Embase alone.

Table 2 shows the number of reviews where we were able to retrieve all the included references. The table also shows the number of reviews where we were able to find 50% or fewer of the included references, and the range of percent of indexed references in the reviews and the range of the indexing rates per category. In these calculations, we excluded the reviews that had no references included (n = 16), as they made the numbers artificially high.

**Table 2:** The span in the indexing rate, per category

Category	No. of reviews with one or more reference included	No. of reviews with 100% of the references indexed in MEDLINE	No. of reviews with 100% of the references indexed in MEDLINE or Embase	No. of reviews with 50% or less of the references indexed in MEDLINE or Embase	Range of indexing rates
Health systems and organization of care	32	19	21	1	17-100%
Health promotion and preventive medicine	47	23	24	3	0-100%
Physical health - treatment	83	50	64	1	0-100%
Physical health - diagnostics	19	10	15	0	63-100%
Mental health - treatment	34	11	19	0	67-100%
Mental health - diagnostics	4	1	1	0	88-100%
Welfare	25	5	5	8	0-100%
Other	14	4	5	1	0-100%
Total	258	123 (48%)	154 (60%)	14 (5%)	

Hence, all of the numbers in this table are based on 258 of the 274 included reviews.

In this sample, the combination of MEDLINE and Embase retrieves the highest number of references. 60% of the reviews have all references indexed in these databases. The numbers for MEDLINE alone are lower (48%). On the other hand, reviews where none of the included references were indexed in MEDLINE or Embase appear in half of the categories. There is a large range of percentages of indexed references in most of the categories, but each category has at least one review where all included references are indexed in either MEDLINE or Embase. The reviews where the indexing rate was 50% or lower totaled 14 (5%). The percentage of indexed references was above 50% in all reviews in the categories “Physical health - diagnostics”, “Mental health - treatment”, and “Mental health - diagnostics”. In these categories, the lowest rates of indexed references were respectively 63%, 67%, and 88%.

As a supplementary analysis, we briefly looked at the 462 non-indexed references. Table 3 shows the publication format of these references. Of the references not indexed in MEDLINE or Embase, 257 (56%) were non-journal articles. These were conference abstracts (n=22), theses (n=38), and other (n=197)). “Other” included health technology assessments and reports, guidelines, and book chapters. Reviews on topics that were not strictly health related more frequently had a high non-indexed-to-included ratio. For example, one review on policing [10] included 40 publications, of which only one was indexed in Embase. Another review on welfare-to-work programs [11] included 41 publications, one of which was indexed in both databases.

**Table 3:** Publication format of non-indexed references

Publication format	Number of references
Journal article	205 (44%)
Conference abstract	22 (5%)
Thesis	38 (8%)
Other	197 (43%)
Total	462

## DISCUSSION

In this project, we investigated reviews produced by NOKC and NIPH and measured how many of the included references were indexed in MEDLINE and Embase. Our results suggest that the majority of references appear in these two databases. Based on this material, we would not recommend changing the current practice and reducing the number of databases to be searched, despite the high indexing rate in MEDLINE and Embase. The fact remains that there are references we would miss by not searching more extensively. Furthermore, while the total indexing rates in the categories of “Health promotion and preventive medicine” and “Physical health - treatment” were high overall (92.1% and 97.4%, respectively), the range of the percentage of indexed references in the reviews was 0-100%. In other words, there are reviews in these categories where we could not retrieve any of the included references. Relying on MEDLINE or Embase alone in those instances would not suffice.

In some of the categories, the indexing rate is considerably lower. “Welfare” had a total indexing rate of 58.9% and the undefined category with the collective name “Other” had 82.5%. There were a few examples of reviews that do not have any health focus at all [10, 11]. Typically, reviews in both these categories had some kind of health aspect to them, though they might have a wider scope and include aspects from other disciplines, like social sciences or education. Given that the health aspect was present, it would theoretically make it more likely that some of the included references were indexed in MEDLINE or Embase. Where the indexing rate was low, it may be that some authors chose to publish outside of medical journals. An example of this can be found in a review on activities offered in senior centers [12], which is in the “Welfare” category. This review has an article published in the journal *Activities, Adaptation, & Aging* (ISSN: 0192-4788) among the included references [13]. Though the article is health-related, the journal has a wider scope, and it is not indexed in either MEDLINE or Embase. Another potential reason for a lower indexing rate is geographical focus. NOKC and NPHI have produced five reviews on female genital mutilation/cutting [14–18], all placed in the “Other” category. The reviews include several references to articles published in African journals. The absence of these journals suggests that, though Embase and MEDLINE are international databases, they may have a European and North American focus and index journals from other parts of the world to a lesser extent.

Reviews that are not strictly health related increase the number of references not indexed in MEDLINE or Embase. Regardless of this fact, 104 of 274 of the reviews included references that were not retrievable in either MEDLINE or Embase. This includes many reviews where the topics are well within the main scope of the databases. In those cases, the publication format is one reason why references are not indexed in these databases. 56% of the references not indexed in the databases are non-journal articles, which were less likely to be found in either database. Only a selection of studies published as reports are included in PubMed, thus making them retrievable in MEDLINE, and neither of the databases indexes theses. But there were also quite a few examples of references that are journal articles on health or medical topics, were published in health or medical journals, and originated from a European or North American country. In those instances, the reason is simply that the databases do not index the journal, at least not in full. Reasons for not indexing a particular journal are only known to the databases themselves.

In contrast to the “Welfare” and “Other” categories, we did not find a similarly low indexing rate in the mental health categories. They were just above average in our results with a total indexing rate of 94.8% (“Mental health - treatment”) and 90.7% (“Mental health - diagnostics”). The range of indexing rates for these categories were narrow, respectively 67-100% and 88-100%. These high indexing rates, combined with the narrow ranges, prompt the question of whether utilizing databases for a specific field of study (like PsycINFO) are worth the extra effort when searching for mental health topics. Considering that “Mental health - diagnostics” was the category with the lowest number of included references, our sample is not large enough to draw a conclusion.

If we were to choose only one database to search, Embase would be the better option, with a slightly higher retrieval rate than MEDLINE. But the highest retrieval rate is achieved by searching the two databases separately. An interesting finding from our data is that even though MEDLINE records are accessible through Embase, Embase cannot fully replace MEDLINE. There are books and reports from NCBI Bookshelf and articles from PubMed Central (PMC) that are searchable through Ovid MEDLINE when choosing the most complete segment, Ovid MEDLINE (MEDLINE ALL), where unique PubMed records are retrievable. These records are not included in Embase.

This study contributes to a conclusion as to whether we can reduce the number of databases to be searched for a systematic review. The next step would be to check if the conclusions in the reviews would differ if the references not found in these databases were not included in the reviews. Halladay et al. [4] and Hartling et al. [19] are examples of studies that have taken the investigation a step further to shed light on this matter. Halladay et al. [4] checked if excluding records not found in PubMed affected the results of 50 Cochrane reviews on the effects of therapeutic interventions. They found that the conclusions did not differ much, concluding that the gains from searching beyond PubMed were modest. Hartling et al. [19] came to a similar conclusion in their study of included references in 129 Cochrane reviews. Effect estimates changed in a minority of meta-analyses and, in most, the change was small, suggesting that selective searching may not introduce bias in terms of effect estimates.

Searching for known articles one by one is an academic exercise that proves that the records are retrievable in theory. In practice, we retrieve the references by constructing a search strategy consisting of subject headings from controlled vocabularies and free-text terms. Even though a reference is indexed in MEDLINE for instance, we do not know if the original MEDLINE search strategy was sensitive enough to retrieve the reference. The reference might have been found in another database, or by other retrieval methods such as citation searching or reference checking. It would be a valuable contribution to the evidence to investigate if the original search strategies are sensitive enough to retrieve all the relevant records in the selected databases.

Our study has a limitation worth noting. Even if a record was retrievable in the databases at the time we carried out this study, this might not have been the case when the search for the review was originally performed. While some articles are indexed as soon as they are published, for others there may be a long lag before they are included. Evaluating searching in selective sources in a prospective manner would be a beneficial contribution to the research on this field.

Our research demonstrates a relative high level of indexing rate in both MEDLINE and Embase on medicine and health-related topics. Both Embase and MEDLINE include unique records and the highest indexing rate is achieved by searching both databases, rather than the complete segment of Embase. There is insufficient evidence to justify changing the practice of searching several databases when performing literature searches for a systematic review at this time, but the data collected in this project provides a good foundation for further investigation.

## Data Availability

Data associated with this article are available in the Norwegian Institute of Public Health Open Repository at https://hdl.handle.net/11250/2977075.
